# TEACCH-based group social skills training for children with high-functioning autism: a pilot randomized controlled trial

**DOI:** 10.1186/1751-0759-7-14

**Published:** 2013-10-01

**Authors:** Kayoko Ichikawa, Yoshimitsu Takahashi, Masahiko Ando, Tokie Anme, Tatsuro Ishizaki, Hinako Yamaguchi, Takeo Nakayama

**Affiliations:** 1 Department of Health Informatics, Kyoto University School of Public Health, Yoshida-Konoe, Sakyo, Kyoto 606-8501, Japan; 2 Center for Advanced Medicine and Clinical Research, Nagoya University Hospital, 65 Tsurumai-cho, Syowa-ku, Nagoya, Aichi 466-8560, Japan; 3 International Community Care and Life-Span Development, Graduate School of Comprehensive Human Sciences, University of Tsukuba, 1-1-1 Tennodai, Tsukuba, Ibaraki 305-8575, Japan; 4 Tokyo Metropolitan Institute of Gerontology, 35-2 Sakae-cho, Itabashi-ku, Tokyo 173-0015, Japan; 5 Osaka Psychiatric Medical Center, 3-16-21 Miyanosaka, Hirakata, Osaka 573-0022, Japan

**Keywords:** Randomized controlled trial, Autism, Social skills training, TEACCH (Treatment and Education of Autistic and related Communication Handicapped Children)

## Abstract

**Background:**

Although social skills training programs for people with high-functioning autism (HFA) are widely practiced, the standardization of curricula, the examination of clinical effectiveness, and the evaluation of the feasibility of future trials have yet to be done in Asian countries. To compensate for this problem, a Japanese pilot randomized controlled trial (RCT) of the Treatment and Education of Autistic and Related Communication Handicapped Children (TEACCH)-based group social skills training for children with HFA and their mothers was conducted.

**Methods:**

Eleven children with HFA, aged 5–6 years, and their mothers were randomly assigned to the TEACCH program (n=5) or a waiting-list control group (n=6). The program involved comprehensive group intervention and featured weekly 2-hour sessions, totaling 20 sessions over six months. The adaptive behaviors and social reciprocity of the children, parenting stress, and parent–child interactions were assessed using the Strengths and Difficulties Questionnaire (SDQ), Parenting Stress Index (PSI), Beck depression inventory-II (BDI-II), and Interaction Rating Scale (IRS).

**Results:**

Through this pilot trial, the intervention and evaluation of the program has been shaped. There were no dropouts from the program and the mothers’ satisfaction was high. The outcome measurements improved more in the program group than in the control group, with moderate effect sizes (SDQ, 0.71; PSI, 0.58; BDI-II, 0.40; and IRS, 0.69). This pilot trial also implied that this program is more beneficial for high IQ children and mothers with low stress than for those who are not.

**Conclusion:**

We have standardized the TEACCH program, confirmed the feasibility of a future trial, and successfully estimated the positive effect size. These findings will contribute to a larger trial in the future and to forthcoming systematic reviews with meta-analyses.

**Trial registration:**

UMIN000004560

## Background

Children with high-functioning autism (HFA) are included within the autism spectrum disorders axis and within normal ranges of intelligence and language functioning, but have impaired cognitive functioning, social skills, and adaptive behaviors. One of the core symptoms of HFA is difficulty in developing friendships due to deficits in sharing emotional experiences and understanding others’ perspectives [1]. Behavioral interventions such as social skills training (SST) can improve social skills, enhance social reciprocity, and promote play skills with peers [2,3]. Previous studies report that many parents of children with HFA experience stress due to their child’s deficits in social communication skills and problem behaviors [4,5]. It was also shown that the less a child’s developmental progress, the more the parents felt stress. Therefore, parent-mediated intervention and assessment of stress levels is critical in any psychosocial intervention [5].

The National Autism Center’s National Standards Report, based on a comprehensive review [6], recognized the Treatment and Education of Autistic and related Communication Handicapped Children (TEACCH) program as a major comprehensive psychosocial program. TEACCH was developed to support people of all ages with autism [7] and features the following two aspects: Structured teaching based on the learning styles of children with autism and teaching parents how to assess and implement individualized support for their children. The TEACCH approach also highlights "fun with others" for developing social skills [8], the goal of which is to acquire social skills, and experiencing positive feelings with other people to improve social reciprocity.

A recent Cochrane systematic review on SST in individuals with autism aged 6 to 21 years [9] identified only five randomized controlled trials (RCTs) and concluded that more RCTs are needed, especially involving individuals aged 7 years or less in a broader spectrum of cultural settings not restricted to the United States. As for TEACCH, the sole RCT that exists at present targeted only parents with autistic children aged 2–3 years without measuring social communication or child adaptive behaviors [10].

Although SST programs or the TEACCH program for people with HFA are widely practiced in clinical or classroom settings in Japan, neither standardization of the curricula nor examination of the effectiveness has as yet been done [3]. This may be due to difficulties in recruiting a sufficient number of participants, especially preschool children, for a clinical trial and to the underdevelopment of relevant clinical research, including RCT, in Japan. However, it is critically important to perform an appropriately designed RCT beginning with the first patient, both from the scientific and ethical viewpoints [11].

Thus, we conducted this pilot RCT to develop a TEACCH-based group SST program for Japanese children aged five to six years with HFA for the following purposes: To standardize the curricula and outcome measures, to identify the characteristics of persons for whom this program was effective, to evaluate the feasibility of a future trial, and to estimate the effect size of this program.

## Methods

### Participants and recruitment

Participants were recruited by mail and telephone from a group including all children and their families who were assessed at a Japanese medical center that specializes in autism and who were eligible according to the inclusion criteria below. We obtained informed consent from the families who joined the study.

Children aged five to six years (one year before entering elementary school) whose diagnoses were autism spectrum disorders confirmed by child psychiatrists (International Statistical Classification of Diseases and Related Health Problems (ICD-10): F84.0), normal intelligence (IQ ≥75), and moderate severity of autism characteristics (CARS-TV ≥25) were included in the study. Exclusion criteria were as follows: 1) Children with severe psychiatric comorbidities (e.g., obsessive-compulsive disorder, conduct disorder, oppositional defiant disorder); 2) Mothers with mental illness with a major obstacle in daily life (e.g., schizophrenia, severe depression, or drug or alcohol dependency).

### Interventions

TEACCH-based group SST program. The TEACCH approach has been widely practiced in Japan since the 1980s, both in clinical and educational settings. The present program was one that had been done in a specialized autism medical center in Osaka, Japan. The program focused on both children with HFA and their mothers. The program involved comprehensive group intervention in the center and featured weekly 2-hour sessions, totaling 20 sessions over six months. The program providers were two psychologists who were trained in TEACCH methodology and had over five years experience, two nursery teachers, one with one year and the other with 15 years of experience with the TEACCH program, two social workers, and two graduate students studying psychology. A psychologist, who served as advisor for the program for over 20 years, supervised the intervention.

Under the program, children were separated from their mothers. An investigator in this study (KI or HY) observed the program from the next room through a one-way mirror and monitored the program by video. The investigator was not directly involved in the program or outcome evaluation. Each session of the children’s program followed the same format:

• Warm-up: To monitor the children’s condition (20 min)

• Description of the rules: To confirm the rules of the program and present the day’s schedule (20 min)

• Game: To practice playing with other children and to learn to understand the rules and the feelings of other children (10 min)

• Social Skills Training: See Table 1 (30 min)

• Exercise: To relax (10 min)

• Reward and feedback: To provide feedback and strengthen learning (20 min)

• Feedback to mothers: To understand their children’s characteristics (10 min)

**Table 1:** Sample activities of social skills training (for children) and outline of mothers’ program

**Sample activities of social skills training (for children)**		
**Session number**	**Session topics**	**Sample activities**^**†**^
1,14,15,20	Pragmatic speech skills	Knowing how to introduce themselves in public, such as vocal volume and the manner of standing.
2,11	Problem solving skills	Knowing how to request assistance or help from other people. Setting a difficult scene and learning the general rules of socially appropriate behaviors by using videotape.
3,4,5,6,	Friendship skills	Understanding the time, inviting to play, borrowing things, refusing a request politely and responding to rejection when a request is refused.
7,8,9	Self-regulation skills	Understanding feelings, such as knowing and controlling emotions when they lose a game, dealing with desires.
10,12,13,16	Nonverbal communication skills	Knowing the feelings of friends, such as practicing facial expressions in a mirror or face-to-face and asking about the feelings and emotions of others.
17,18,19	Shopping skills	Knowing shopping rules and paying with money at the cafe.
**Outline of mothers’ program**		
**Session number**	**Session topics**	
1	Introducing themselves and understanding the concepts of the program.	
2,6,9,13,19	Watching the children’s program through a one-way mirror, understanding the TEACCH method and joining SST with their children.	
3	Learning about development of HFA from a child psychiatrist.	
4,7,8	Setting a goal for daily life, individual consultation by social workers and peer counseling.	
5,10,11,12,14,18	Learning parent training, such as making manuals for their children or using effective instructions and communication with positive feedback.	
15,16,17	Consulting about educational support for elementary school, and making a support system and a "support-book".	
20	Joining the farewell ceremony and practicing positive feedback with their children.	

^†^All sessions consisted of instruction, video modeling, rehearsal, feedback and demonstration of aspects of the real world.

Table 1 shows sample activities in the SST program [2] and outlines the mothers’ program. Each program cost approximately 800 Japanese Yen (about 10 US dollars), along with a payment from health insurance (the total cost for the whole program was approximately 200 US dollars).

Waiting-list control group. Group meetings for mothers were held three times (once every two months) with two social workers for 30–60 minutes per session. The contents of the meetings were not specified or directed by the investigators. After a 6-month non-intervention period, the TEACCH-based SST program was also provided to the control group.

### Measures

Baseline demographic characteristics included the children’s profiles and family characteristics: The child’s age, sex, IQ, severity of autism, social competence, disabilities of their siblings, experience in special education programs, mothers’ age, profession, parents’ level of education, use of medical subsidies for infants and children, and educational support for children. DQ was measured by K-test [12], which was equivalent to the IQ of younger PDD children [13]. The severity of autism was measured by the Childhood Autism Rating Scale-Tokyo version (CARS-TV) **[**[14,15]. Social competence was measured by the Japanese version of the Social Maturity Scale (SQ: social quotient) [16], which was correlated with those of the Vineland Adaptive Behavior Scales (VABS) [17].

Primary outcome: SDQ. Strengths and Difficulties Questionnaire (SDQ) [18] measures the adaptive behaviors and social reciprocity of children aged 4–16 years. We used the 25-item version for teachers and parents, which consists of five subscales: Pro-social behavior, conduct problems, hyperactivity, emotional problems, and peer problems [19]. We evaluated the SDQ score provided by the mothers.

Secondary Outcomes: PSI, BDI- II, IRS. The Japanese version of the Parenting Stress Index (PSI) [20,21] measures parental stress in two major domains: Characteristics of the child and parent. The Beck Depression Inventory-II (BDI-II) is a 21-item depression scale for assessing emotional, behavioral, and somatic symptoms [22,23]. The Interaction Rating Scale (IRS) [24], which was developed in Japan, is used to measure a child’s social competence and a caregiver’s child rearing competence via five-minute observations of caregiver-child interactions. In this study, we recorded child and parent interactions while they played with a puzzle: Two independent, trained evaluators who were blind to the allocation evaluated child-mother interaction in the videos.

### Feasibility of a future trial

We examined the feasibility of a future trial by use of questionnaires that assessed the causes of dropout and mothers’ satisfaction with the contents of the program. We also investigated the mothers’ satisfaction with the length of the session, the duration of intervention, and the frequency and cost of the program, as well as any adverse events or burdens associated with the program.

We also described the baseline characteristics of each child in the intervention group to determine the characteristics of suitable participants for this program.

### Study design

Eligible children and their mothers were randomly assigned to an intervention or control group. Children were evaluated both by their mothers and by independent evaluators. The mothers self-reported the condition of their child at baseline and within one month after the end of the intervention (six months after the baseline).

### Randomization & sequence generation, allocation concealment

After baseline assessments, an independent allocation manager ran a computer-generated allocation schedule based on the following three variables based on the minimization method of balancing three variables: Sex, autism severity (CARS score ≤30 or CARS score >30), and social competence (SQ score ≤100 or SQ score >100). Program providers were unaware of the allocation sequences.

To avoid bias, we standardized measurements for assessment and employed independent evaluators and independent allocation managers who were blinded to allocation. However, intervention allocation could not be masked from the children and their mothers in the program or from the program providers.

### Statistical methods

To evaluate the effectiveness of SDQ, PSI, BDI-II, and IRS, we used intention-to-treat (ITT) analysis and analysis of covariance (ANCOVA). We compared the post-intervention scores between the two groups with baseline scores as a covariate. Because of the pilot nature, no hypothesis testing was employed, but 95% confidence intervals (CI) were described for estimated values. Effect size (Cohen’s *d*) was calculated by dividing the baseline SD score of the overall sample by the difference between the intervention and control groups. The sample size required for a future large trial was estimated on the basis of SDQ scores using Stata (version 11.0). Additionally, we analyzed the correlation between the IRS scores of two independent evaluators using Spearman’s rank correlation coefficient. Reporting of the study was complied with the Consolidated Standards of Reporting Trials (CONSORT) statement for non-Pharmacologic Treatment [25,26]. Analyses were performed using SPSS ver.19 after completion of the assessments.

### Ethical standards

The study was approved by the Kyoto University Graduate School and Faculty of Medicine Ethics Committee (Registered number: C493). There were no monetary incentives for the participants to join the program. This study was registered in the University Hospital Medical Information Network Clinical Trials Registry (UMIN000004560).

## Results

**Figure 1:**
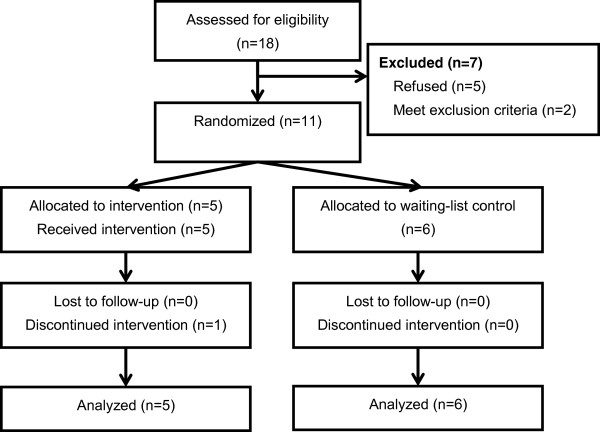
Participant flowchart.

Among 18 families that met the eligibility criteria, five declined to participate given the difficulty of regular participation due to distance (n=2), a subjective burden of the high frequency of the program (n=2), and not feeling the need to participate (n=1). We excluded two families because the children had psychiatric comorbidities: Oppositional defiant disorder. Eleven children were randomly assigned to the program (n=5) or the control group (n=6). Although one family in the program group participated in only 10 sessions due to the mother’s pregnancy, we assessed all participants at the end of the program (see Figure 1).

Demographic features were slightly different between the groups, including age, sex, DQ score, diagnosis, CARS score, SQ score, and background of family education and socio-economic status (see Table 2). Eight families were receiving medical subsidies and five had educational support. Seven families had another child diagnosed with autism.

**Table 2:** Baseline characteristics

**Baseline characteristics**^**†**^	**Intervention (n=5)**	**Control (n=6)**	**Overall (n=11)**
**Children’s resources**			
Age (months)	64 (60–66)	62 (60–70)	64 (60–70)
Median (min-max)			
Sex, Male: Female	4:1	5:1	9:2
Diagnosis: PDD, n	4	2	6
Diagnosis: Asperger, n	1	1	2
Diagnosis: HFA, n	0	3	3
DQ, Median (min-max)	87 (84–117)	88 (78–145)	88 (78–145)
CARS score, Median (min-max)	32.5 (27.5-33.5)	31.0 (26.5-33.0)	30.0 (27.5-33.0)
SQ, Median (min-max)	90 (81–101)	96 (71–105)	96 (71–105)
**Social resources**			
Mother’s Age (years), Median (min-max)	42 (36–45)	36 (33–41)	39 (33–45)
Education level of mother, level (n)	High School (2)	High School (1)	High School (3)
	Junior College (3)	Junior College (2)	Junior College (5)
		University (3)	University (3)
Education level of father, level (n)	High School (1)	High School (2)	High School (3)
	University (4)	University (3)	University (7)
		Unknown (1)	Unknown (1)
Working mother, n	1	2	3
Medical subsidy, n	4	4	8
Education support, n	2	3	5
Siblings with Disabilities, n	3	4	7
Experience with special education programs, n	4	4	8

^†^PDD indicates Pervasive Developmental Disorders; Asperger, Asperger Syndrome and HFA, High Functioning Autism. DQ indicates scores in the "Kyoto Scale of Psychological Development". CARS indicates scores in the "Childhood Autism Rating Scale-Tokyo version". SQ indicates scores in the "Japanese version of Social Maturity Scale".

Attendance in the program was as follows (the control group was assessed after completing the program): Six families completed all 20 sessions, two families completed 19 sessions, one family completed 18 sessions, one family completed 15 sessions, and one family completed 10 sessions (due to the mother’s pregnancy). Regarding program satisfaction, seven families (64%) were satisfied with the overall program, 10 (91%) with the length of each session, nine (82%) with the frequency of the program, eight (73%) with the duration of intervention, and eight (73%) with the cost of the program. Eight families responded that they would recommend this program to others. Nine families responded that they did not have enough time to practice TEACCH in their daily lives. The preferred aspects of the program were "peer counseling (n=11)", "watching the children’s program (n=6)", "parent training (n=6)", and "making manuals for children (n=5)". No adverse events occurred during the program.

**Figure 2:**
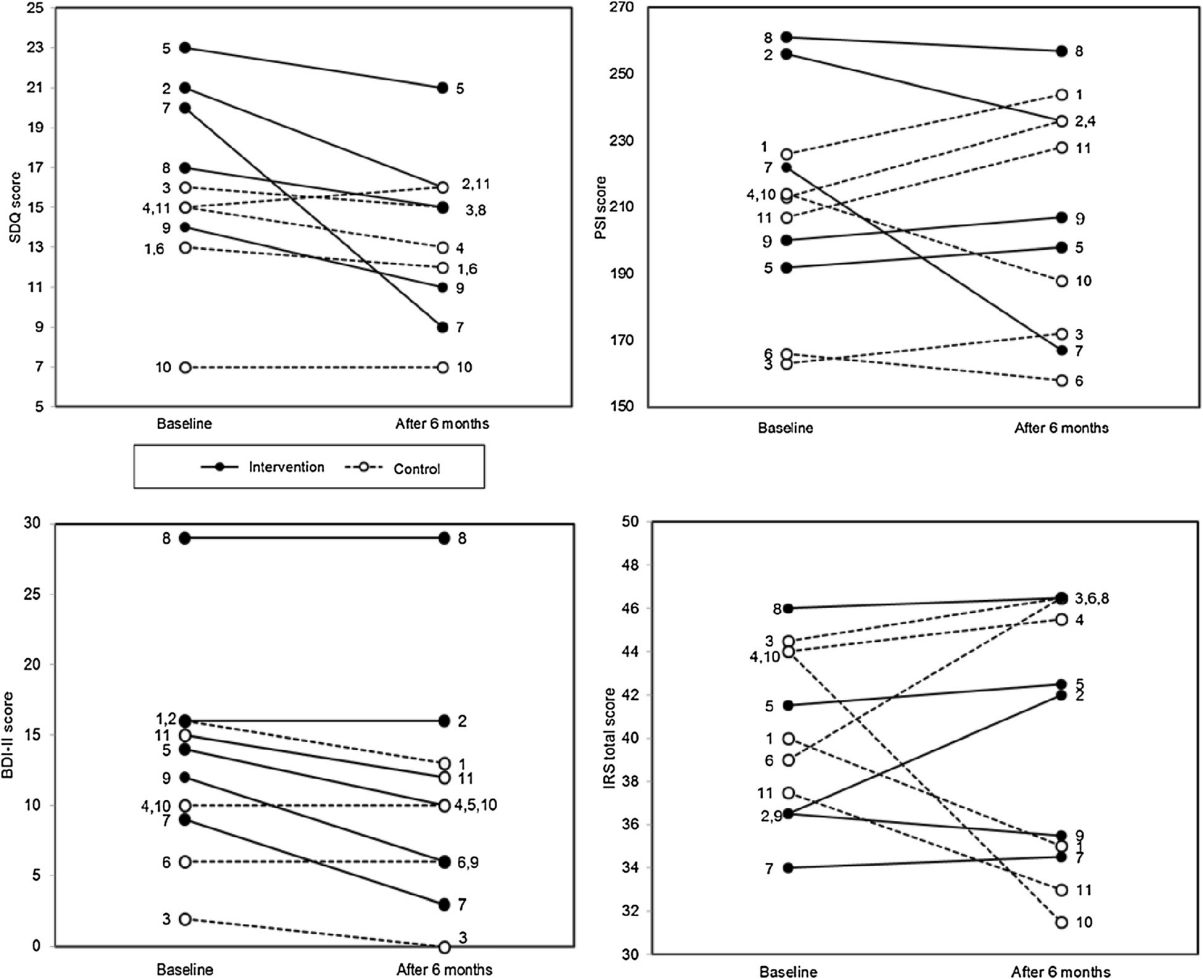
**Change in scores: SDQ, PSI, BDI-II, IRS.** Numbers in the figures show each participant’s ID, SDQ: Strengths Difficulties Questionnaire. 0<normal<12, 13<borderline<15, 16<high risk<40, PSI: Parenting Stress Index. Standard score of the Japanese population is 190.8±29.8 (mean±SD). Larger score indicates a more severe condition, ‧BDI-II: Beck Depression Inventory II. Standard score of the Japanese population is 8.9±6.5 (mean±SD). Larger score indicates a more severe condition, ‧IRS: Interaction Rating Scale. Smaller score indicates a more severe condition.

The difference in SDQ scores between the intervention and control groups at the endpoint was -3.12 (effect size =0.71). The difference in PSI scores was -18.12 (effect size =0.58) and in BDI-II scores was -2.77 (effect size =0.40). The difference in IRS scores (average of two independent evaluators’ scores) was 2.72 (effect size =0.69). Detailed differences in each participant’s score between baseline and endpoint are shown in Figure 2 and Table 3. From the baseline scores of SDQ and BDI-II, five children were designated as high risk, five were designated as borderline with regard to adaptive behaviors, and nine of the mothers exhibited moderate to severe depressive symptoms.

**Table 3:** Means, differences and effect sizes for intervention and control groups

**Scale**	**Point**	**Intervention (n=5)** **Mean (SD)**	**Control (n=6)** **Mean (SD)**	**Difference**^**a**^	**95% confidence interval**	**Effect size (d)**
SDQ	Baseline	19.0 (3.5)	13.2 (3.3)			
	After 6 months	14.4 (4.7)	12.5 (3.2)	-3.12	-8.42, 2.18	0.71
PSI	Baseline	226.2 (31.5)	198.2 (26.8)			
	After 6 months	213.0 (34.8)	204.3 (36.3)	-18.12	-55.75, 19.50	0.58
BDI-II	Baseline	16.0 (7.7)	9.8 5.3)			
	After 6 months	12.8 (10.3)	8.5 (4.8)	-2.77	-6.30, 0.75	0.40
IRS-total	Baseline	38.9 (4.8)	41.5 (3.0)			
	After 6 months	40.2 (5.1)	39.7 (6.0)	2.72	-5.83, 11.27	0.69

^a^Difference: difference of scores between Intervention and Control groups, with baseline scores as a covariate.

SDQ: Strengths and Difficulties Questionnaire, PSI: Parenting Stress Index, BDI-II: Beck Depression Inventory-II, IRS: Interaction Rating Scale.

The characteristics of child (ID7) whose SDQ score was remarkably improved are as follows: High IQ score (DQ 117), mother with low subjective stress, and no siblings with disabilities. The characteristics of child (ID2) whose SDQ score was moderately improved are a mother with low subjective stress and no siblings with disabilities. The characteristics of child (ID8) whose SDQ score was not improved are as follows: Mother with high stress, siblings with disabilities, and female gender. The characteristics of child (ID9) whose SDQ score was not improved are a low attendance rate at the program, siblings with disabilities, and the mother’s low satisfaction with the program. Concerning child (ID5), the effectiveness of intervention was not clear because this child attended only half of the program due to the mother’s pregnancy.

Correlations between the IRS scores of the independent evaluators were ρ=0.70 for the child IRS score, ρ=0.36 for the parent IRS score, and ρ=0.56 for the total IRS score. The required sample size for a future trial was estimated to be approximately 50, allowing for 10% attrition, with a two-sided *p* value of 0.05 and an 80% power calculation.

## Discussion

This is the first RCT to evaluate the TEACCH-based group social skills training program for Asian children aged five to six years with high-functioning autism and to reveal the feasibility and potential efficacy of this program. Koenig reported that "parents wished for more direct feedback with what was taking place in the group sessions in order to follow up with skill-building at home" [27]. We asked the mothers for direct feedback at the end of each session, and they sometimes watched the programs through one-way mirrors. These considerations may have contributed to the high level of satisfaction with the program. However, the mothers who participated in this program did not use TEACCH skills often in their daily lives. Providing materials, homework, or incentives are potential next steps to put TEACCH skills into daily life.

The effect of the program was quite satisfactory with regard to the children’s adaptive behaviors, social reciprocity, parenting stress, and parent–child interactions. Because the sample size required for a future trial was estimated to be approximately 50 participants, recruitment of participants at multiple facilities similar to the present one is required.

Additionally, the characterization of each child in the intervention group suggested that this program was more effective for the participants whose IQ was high or who had no siblings with disabilities, but was not effective when the mother was heavily stressed. Specialized individual programs may be needed for children with stressed mothers or for lower DQ children. The baseline scores of SDQ and BDI-II revealed that children with HFA had more difficulty in adapting to society and that their mothers were more depressed than their general counterparts. The mothers requested that the program be longer and asked for continuous support after the program. This suggests that we should verify the optimum length of the program in future trials to address this issue.

There are some limitations with this study. First was the lack of blindness because the primary outcome (SDQ) was evaluated by participating mothers. To date, there is no objective measure of children’s social skills in Japan. To compensate for this, we used independent rater outcome (IRS) to assess secondary outcomes. High agreement between measurements by two independent evaluators indicated little possibility of serious bias in the present evaluation. The second was limited sample size. The present trial was a pilot of which the primary purpose was not to test a hypothesis but to standardize the program, examine its feasibility, and to estimate potential effect size. Because randomization was strictly conducted and reported in accordance with the CONSORT statement, the present trial was successful and will lead to a relevant larger trial and forthcoming systematic reviews with meta-analyses.

## Conclusion

This pilot study confirmed the feasibility and potential efficacy of TEACCH-based social skills training in Asia. High-functioning children with autism were able to develop social skills and reciprocity, and their mothers’ stress was reduced. This trial also implied that this program may be especially effective for high IQ children and mothers with low stress. It will contribute to a larger trial in the future and to forthcoming systematic reviews with meta-analyses.

## Competing interests

This study was funded by a grant from the Meiji Yasuda Mental Health Foundation in 2011. The foundation had no role in data collection, analysis or interpretation of the study. We declare that we have no conflicts of interest.

## Authors’ contributions

KI conceived this study and performed the study design and the statistical analysis. YT and TI and TN participated in the design of this study and helped to draft the manuscript. MA made the computer based random allocation system. TA developed the Interaction Rating Scale (IRS) and trained the independent evaluators. HY coordinated and observed the intervention to check the feasibility of intervention. All authors read and approved the final manuscript.
